# Biomechanics of selected arborescent and shrubby monocotyledons

**DOI:** 10.3762/bjnano.7.154

**Published:** 2016-11-07

**Authors:** Tom Masselter, Tobias Haushahn, Samuel Fink, Thomas Speck

**Affiliations:** 1 Plant Biomechanics Group, Botanic Garden, Faculty of Biology, University of Freiburg, Schänzlestraße 1, D-79104 Freiburg im Breisgau, Germany

**Keywords:** arborescent monocotyledons, biomechanics, biomimetics, *Dracaena*, functional morphology

## Abstract

Main aims of the study are a deepened understanding of the mechanically relevant (ultra-)structures and the mechanical behaviour of various arborescent and shrubby monocotyledons and obtaining the structure–function relationships of different structurally conspicuous parts in *Dracaena marginata* stems. The stems of five different “woody” monocotyledon species were dissected and the mechanical properties of the most noticeable tissues in the five monocotyledons and, additionally, of individual vascular bundles in *D. marginata*, were tested under tensile stress. Results for Young’s moduli and density of these tissues were assessed as well as the area, critical strain, Young’s modulus and tensile strength of the vascular bundles in *Dracaena marginata*. These analyses allowed for generating a model for the mechanical interaction of tissues and vascular bundles of the stem in *D. marginata* as well as filling major “white spots” in property charts for biological materials. Additionally we shortly discuss the potential significance of such studies for the development of branched and unbranched bio-inspired fibre-reinforced materials and structures with enhanced properties.

## Introduction

**Figure 1:**
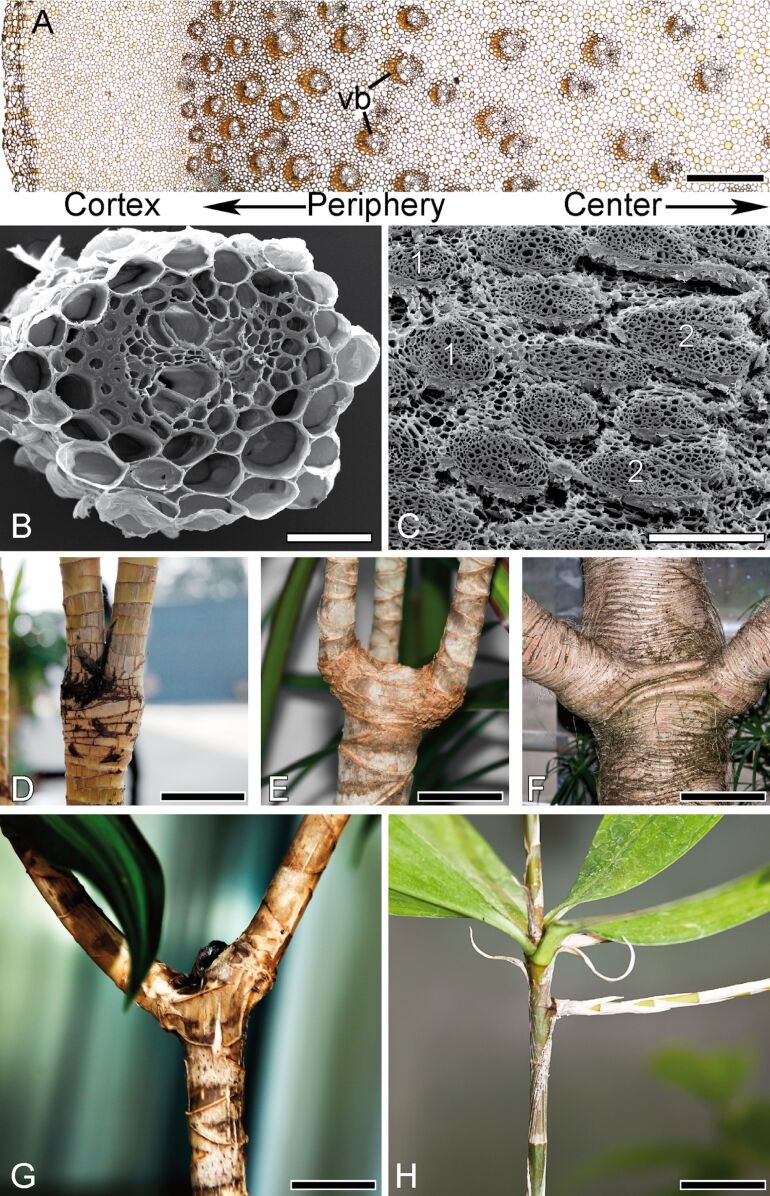
Morphology and anatomy of various monocotyledons. (A) Cross-section of *Dracaena marginata*, showing the dispersed vascular bundles (vb) in the parenchymatous matrix. (B) Cross-section of primary vascular bundle. (C) Cross-section of primary vascular bundles (1) and secondary (amphivasal) vascular bundles (2). (D) Branching morphology in *D. fragrans*. (E) Branching morphology in *D. marginata*. (F) Branching morphology in *Pandanus pygmaeus*. (G) Branching morphology in *D. reflexa*. (H) Branching morphology in *D. surculosa*. Scale bars: (A) 1 mm, (B) 0.1 mm, (C) 0.5 mm, (D) 50 mm, (E) 40 mm, (F) 150 mm, (G) 20 mm, (H) 20 mm.

**Figure 2:**
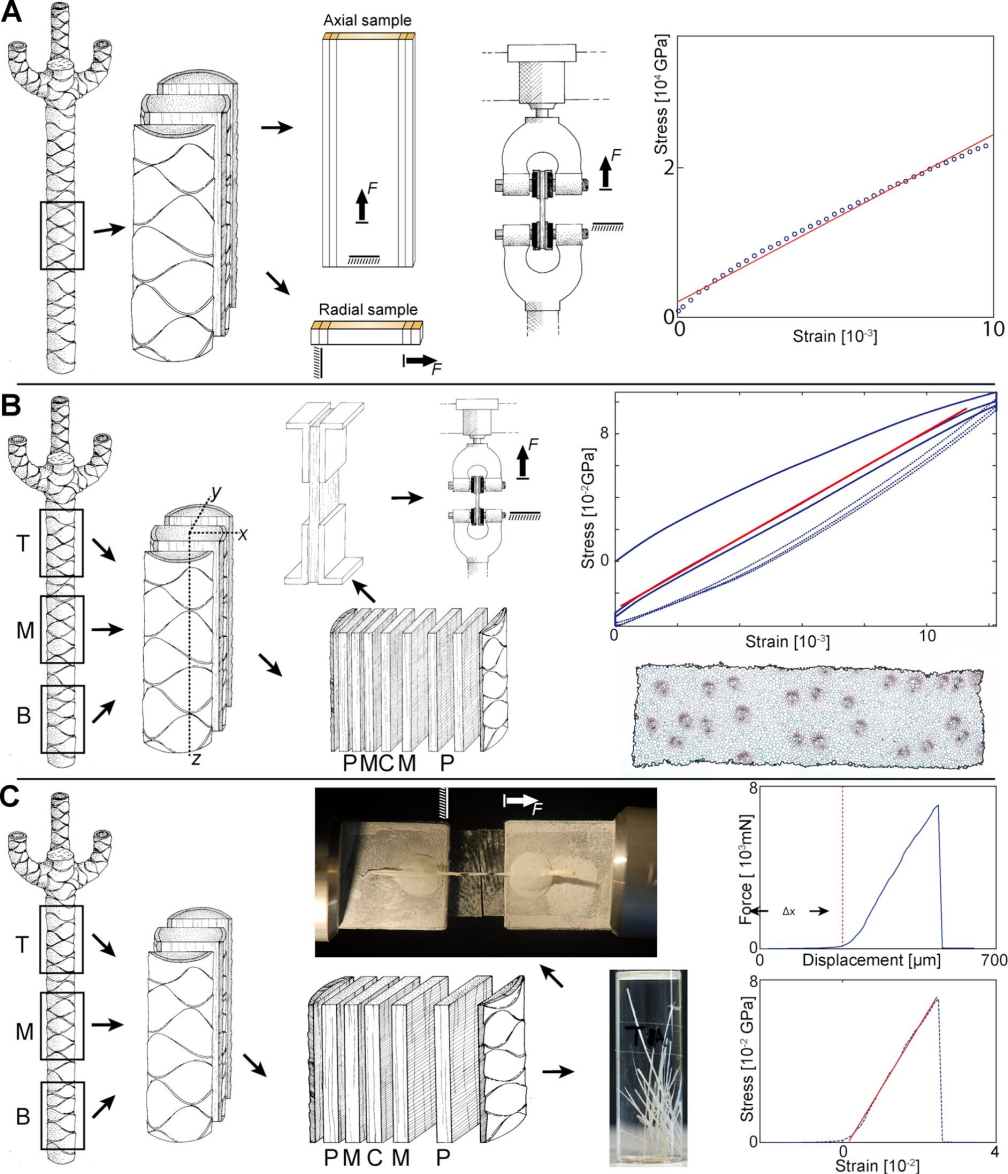
Experimental setups. (A) Setup for measurements of stem samples at various axial positions within the plant. Tensile Young’s moduli were evaluated in radial and axial direction by the OLS (ordinary least squares) slope (red line) of the stress–strain diagram of respective datasets for all five tested monocots. (B) *D. marginata* was investigated in more detail. Samples with radial widths of *x* and *y* as well as an axial length of *z* were taken in this species from previously defined vertical (B, M and T) and radial zones (C, M, P) within the stem. Before testing, samples were fixed with glue to L-shaped aluminium brackets so that the samples could be tested under tensile stress. The evaluation of the Young’s moduli was based on the OLS slope of the second load cycle (red line). Subsequently the cross-section of each sample was determined by analysing images of microtome slides. (C) Vascular bundles from *D. marginata* were extracted from the same defined zones as in (B), and tested until failure. *F* is the force acting on the samples, Δ*x* is the distance until a significant increase in force occurred.

For many centuries, botanists and non-biologists alike have expressed their fascination about the conspicuous growth form of arborescent monocotyledons. Nevertheless, only in the middle of the 20th century first attempts were made to understand the form–structure–function relationships of these plants. To date, while the variation of physical properties from top to base and centre to periphery, as well as the underlying structural features, are well known in many dicotyledonous trees [1], these property shifts are still hardly studied in tree-like monocotyledons. This knowledge deficit is largely caused by a lack of interest for empirical data for monocotyledon stems and is a result of their insignificance as constructional material in many (industrialized) countries with the major exception of bamboo culms [2]. Results for physical properties of dicot plants cannot be transferred to monocots as the organization of dicotyledonous stems is significantly different from that of monocotyledonous stems (Figure 1). The present study allows for closing some of these knowledge gaps and filling major “white spots” in natural material property charts. To this aim, analyses of the mechanical properties concentrate on two hierarchical levels: On a first level, the radial and axial Young’s moduli of stem tissues in the five “woody” monocotyledon species are analysed (Figure 2A). In addition, in stems of *Dracaena marginata*, which was chosen as a representative model plant for “woody” monocotyledons (see below), the variations of the axial Young’s modulus and the tissue densities at different radial and axial positions are assessed (Figure 2B). On a second hierarchical level, the Young’s moduli and the tensile strengths of individual fibrous vascular bundles of *D. marginata* are investigated (Figure 2C). This enables a direct assessment of the contribution of the fibrous bundles to the mechanical properties of the underlying tissue by applying the rule of mixture. This procedure promises a considerable improvement to most studies that take a reverse indirect approach and extrapolate fibre properties by using the fibre volume fraction in the tissue [3–5]. Finally, the results for the mechanical properties of both tissues and fibrous vascular bundles are compared to available data from dicotyledonous and monocotyledonous stems.

The obtained data provide the basis for follow-up investigations on the branching mechanics of “woody” monocots. In addition, these data can be incorporated in finite element models at cell and tissue level that mirror the anisotropy and the stress–strain behaviour of the investigated plants at stem level [6–7]. This allows for a deepened understanding of the structural and mechanical requirements of *Dracaena marginata*, which was chosen as a representative model organism for arborescent monocotyledonous plants with lignified vascular bundles and anomalous secondary growth.

## Morphology and anatomy of monocotyledons

The model plant *Dracaena marginata* was chosen for a generalized anatomical description (Figure 1A–C,E) as the morphology and anatomy of the other four monocotyledons analysed – *D. fragrans* (Figure 1D)*, D. reflexa* (Figure 1G), *D. surculosa* (Figure 1H) and *Pandanus pygmaeus* (Figure 1F) – are very similar while differing in detail. Monocotyledon shoots are organized in an atactostele (Figure 1A), which means that individual vascular bundles with sclerenchymatous caps are dispersed irregularly within the ground tissue matrix (parenchyma) of the stem. The central cylinder is a multi-gradient structure in terms of size of vascular bundles (decrease towards periphery), number of vascular bundles (increase towards periphery) and cell size of the parenchymatous ground tissue (decrease towards periphery). These gradients are well visible in a cross-section of *D. marginata* (Figure 1A). Limited to a small group of monocotyledons, amongst these *D. marginata*, secondary vessels (Figure 1C) are formed at the border between the central cylinder and the surrounding cortex.

## Results

### 1 Young’s modulus of five different monocotyledons

**Figure 3:**
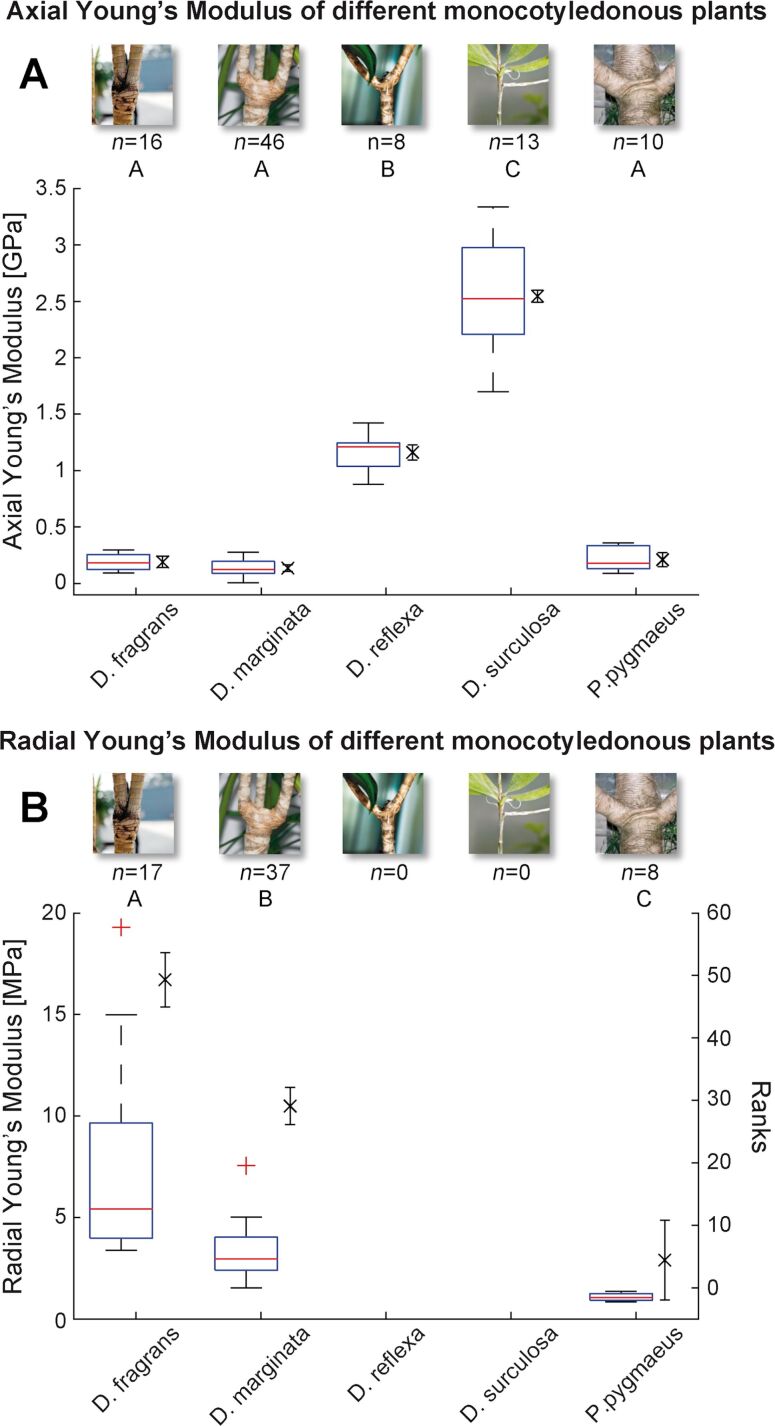
Axial (A) and radial (B) Young’s moduli for *Dracaena fragrans*, *D. marginata*, *D. reflexa*, *D. surculosa* and *Pandanus pygmaeus*. Significances on the 5% level (group means (A), mean ranks (B)) are displayed in letter grouping, where identical letters group data with no significant difference. The number of tested samples is indicated by *n*. In both A and B, the full range of data is visualized by a boxplot of medians, quartiles (box), extreme values (whiskers) and potential outliers (+). In addition, the mean (×) and standard error (whiskers) are plotted as error-bar plot in A, whereas in B the mean ranks (×) and standard error (whiskers) are plotted as error-bar plot.

Results for the Young’s modulus from experimental setup one (see paragraph 1 in section ’Experimental’) after measurements in axial direction are given in Figure 3A and in Supporting Information File 1 – Raw data. The box–whisker plots indicating median, interquartile range and extreme values show the range of values of the Young’s modulus found for the different monocot species tested. The min–max ranges are 0.2017 GPa for *Dracaena fragrans* (median of axial Young’s modulus: 0.1799 GPa), 0.2711 GPa for *D. marginata* (median of axial Young’s modulus: 0.1235 GPa), 0.5445 GPa (median of axial Young’s modulus: 1.2090 GPa) for *D. reflexa*, 1.6386 GPa for *D. surculosa* (median of axial Young’s modulus: 2.5217 GPa) and 0.2697 GPa for *Pandanus pygmaeus* (median of axial Young’s modulus: 0.1790 GPa). The normality assumption is not rejected on the basis of a Lilliefors test and the data are therefore additionally presented by mean and standard error of the mean for each plant respectively (see Supporting Information File 1 – Descriptive statistics). An ANOVA with post-hoc multiple comparison (with Bonferroni correction) on means shows no significant differences for the tree-like monocot species *D. marginata*, *D. fragrans* and *P. pygmaeus*. On the other hand, *D. surculosa* (shrub-like) and *D. reflexa* (tree-like) differ significantly (on the 5% level) from each other and also from the three other tree-like species (ANOVA; F_4,88_ = 427.33, *P* less than 0.001, see Supporting Information File 1 – Inferential statistics). The significances are shown in Figure 3 by letter grouping, where the same letter indicates no statistically significant difference. Notable is the significantly higher axial Young’s modulus found in the shrub-like *D. surculosa.*

Measurements of the Young’s modulus in radial direction (Figure 3B, see Supporting Information File 1 – Raw data) show in two tested species (*D. fragrans* and *D. marginata*) a wide range of values (min–max range of 0.0159 GPa for *D. fragrans* (median of radial Young’s modulus: 0.0054 GPa), 0.0060 GPa for *D. marginata* (median of radial Young’s modulus: 0.0029 GPa), and 0.0005 GPa for *Pandanus pygmaeus* (median of radial Young’s modulus: 0.0010 GPa), which are visualized by a box–whisker plot of the data. As the assumption for normality (Lilliefors test) was rejected for *D. fragrans*, here the radial Young’s moduli are additionally described with mean ranks and respective errors of the mean ranks. A Kruskall–Wallis test with post hoc multiple comparison shows significant differences (at 5% level) (chi-squared = 35.12, 2, *P* less than 0.001) between all measured plants, indicated in Figure 3B with letter grouping as described above (see Supporting Information File 1 – Inferential statistics). No results were obtained for *D. reflexa* and *D. surculosa* due to small radial dimensions of the stems that prohibited measurements. Values of axial and radial Young’s moduli differ in all tested species by a factor between 7.46 and 171.

### 2 Young’s modulus and density of stem samples in relation to the radial and axial position in *Dracaena marginata*

**Figure 4:**
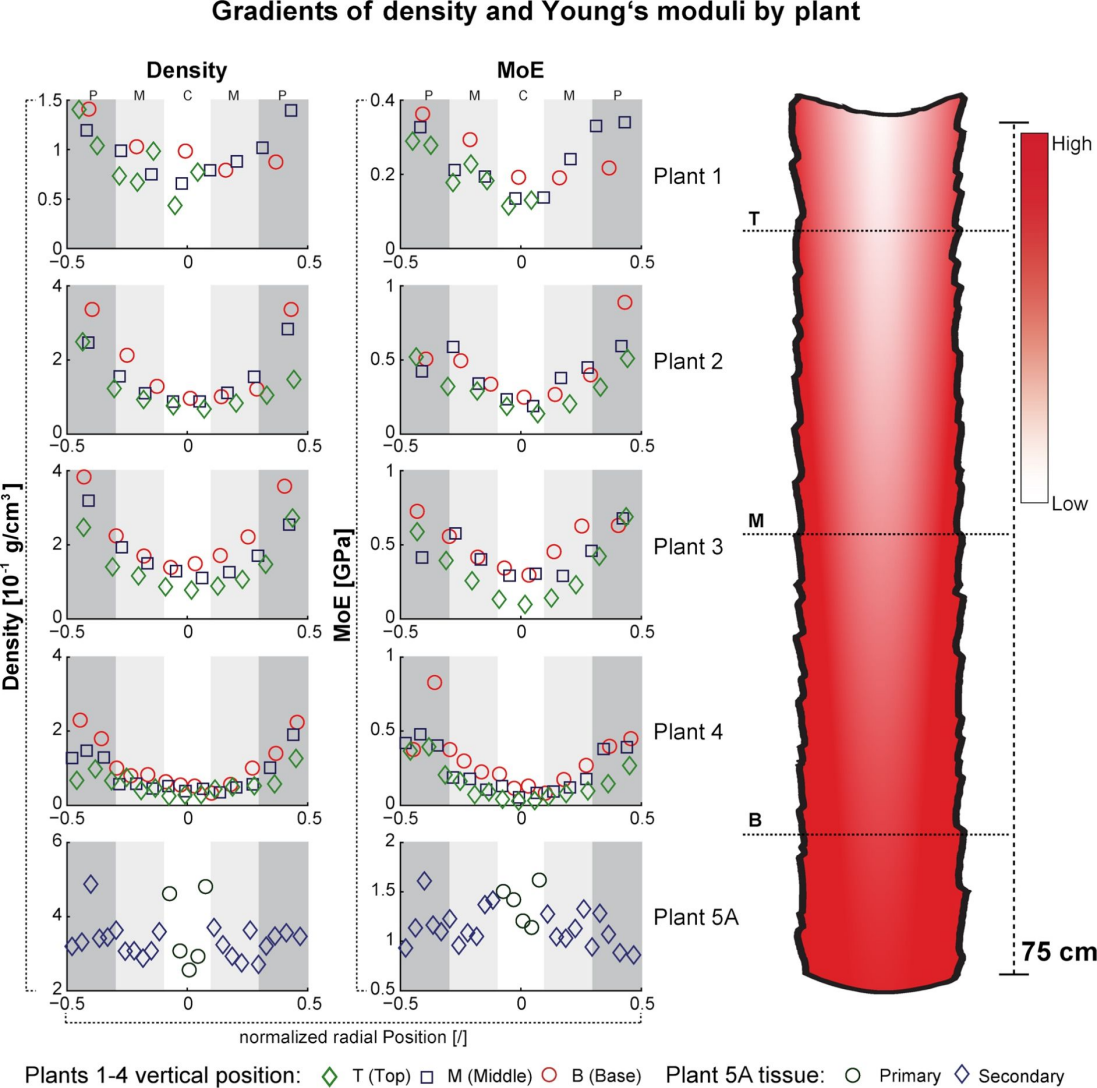
Dry density and Young’s modulus (MoE) in five stem samples of *Dracaena marginata*. In radial positions from stem centre (C, white area) over middle position (M, light grey area) to stem periphery (P, dark grey area) shown by normalized radial position and categorized by vertical position, i.e., base (B), middle (M) and top (T) level of the respective stems. Plants 1–4 possess only primary tissue. In plant 5 set A, data are shown for primary and secondary tissues. Note the U-shaped data distribution of dry density and MoE for plants 1–4 and for the primary tissues of plant 5A. Also note the broad scatter in density and MoE across the diameter for the secondary tissue in plant 5A.

In Figure 4, density and axial Young’s modulus are displayed for the different axial zones (basal (B), middle (M) and top (T)) and radial zones (periphery (B), middle (M) and centre (C)) of the tested plants, for the stems below branchings (plants 1–4) and a first order branch (plant 5). Plants 1 to 4, in which all samples consisted only of primary tissue, show a distinct U-shaped pattern for values of density and Young’s modulus over the stem diameter for all three axial zones. It can be observed that the lowest value for density and Young’s modulus (MoE) always occur in the radial centre of the stem and the values increase towards the periphery. In addition, the samples for these four tested plants show a similar trend in axial direction. The highest values, for density as well as for Young’s modulus, occur at the stem base and decrease apically towards the top of the stem. This results in a twofold gradient within the plant stem (Figure 4). For plant 5, which holds a high amount of secondary tissue, the data for density and Young’s modulus of primary and secondary tissues show a broad scatter across the diameter with no distinct tendency. Additionally, plant 5 shows a higher density and MoE as plants 1–4. Overall, the density lies in a range from 0.025 to 0.487 g/cm^3^ with a mean value of 0.171 ± 0.11 g/cm^3^, and the axial Young’s modulus lies in a range between 0.03 and 1.62 GPa with a mean value of 0.52 ± 0.39 GPa.

**Figure 5:**
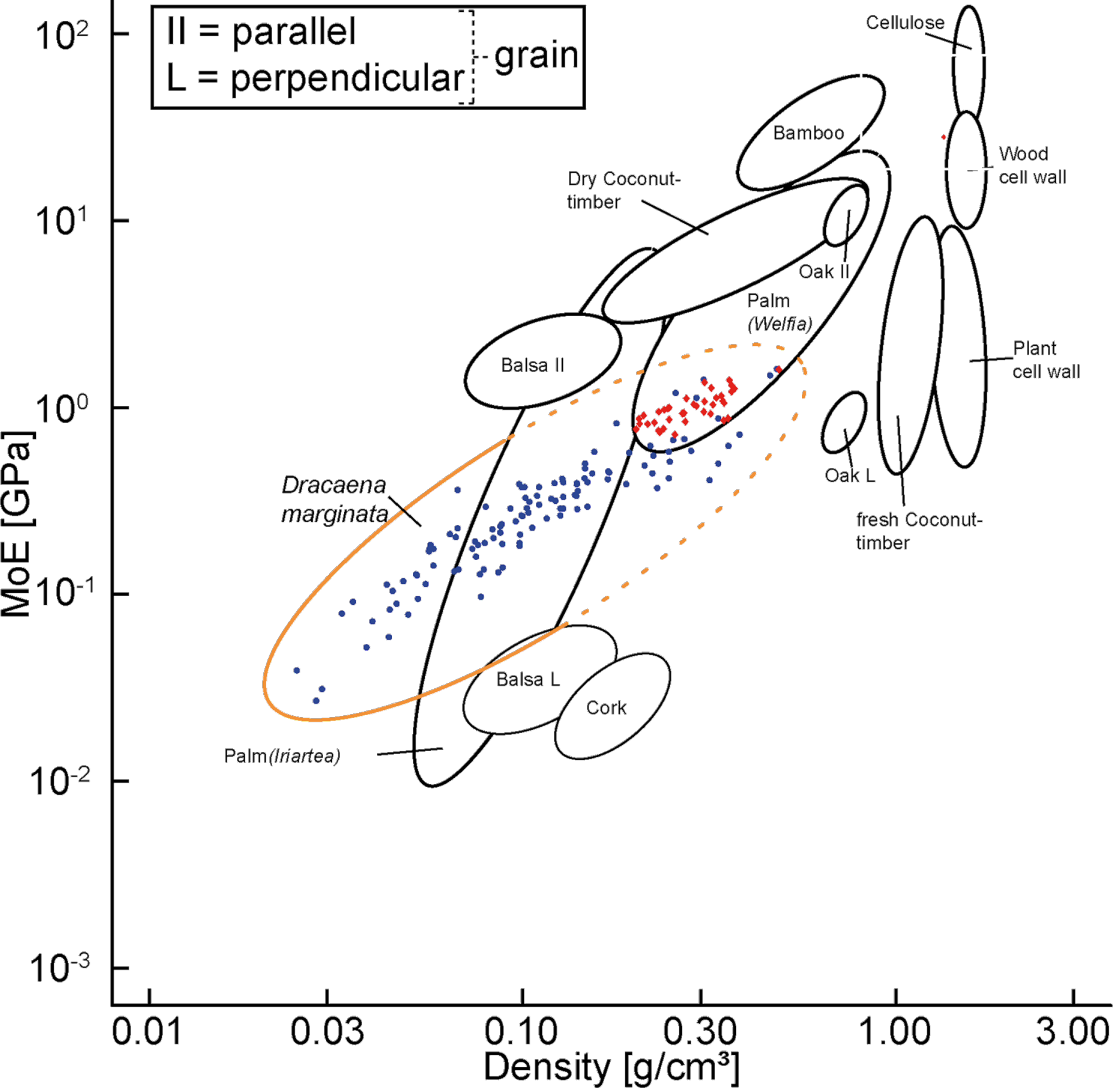
Materials property chart with measured values for stem samples of *Dracaena marginata*. Blue: primary tissues, red: secondary tissues in the log–log scale of Young’s modulus (MoE) vs density. Samples tested parallel to the grain/vascular bundles (adapted from [9]).

Figure 5 shows the relationship between density and axial Young’s modulus of the tested tissue samples of *D. marginata* on a log–log scale in a materials property chart in relation to other plant species and materials. A statistically significant exponential correlation exists between density and axial Young’s modulus as well for primary as for secondary tissue (linear model (OLS): F_4,148_, *P* less than 0.001, see Supporting Information File 2). It can also be seen that *D. marginata* covers a wide range of values for density and Young’s modulus, a pattern that was also found for palms [8].

### 3 Tests of the vascular bundles of *Dracaena marginata*

**Figure 6:**
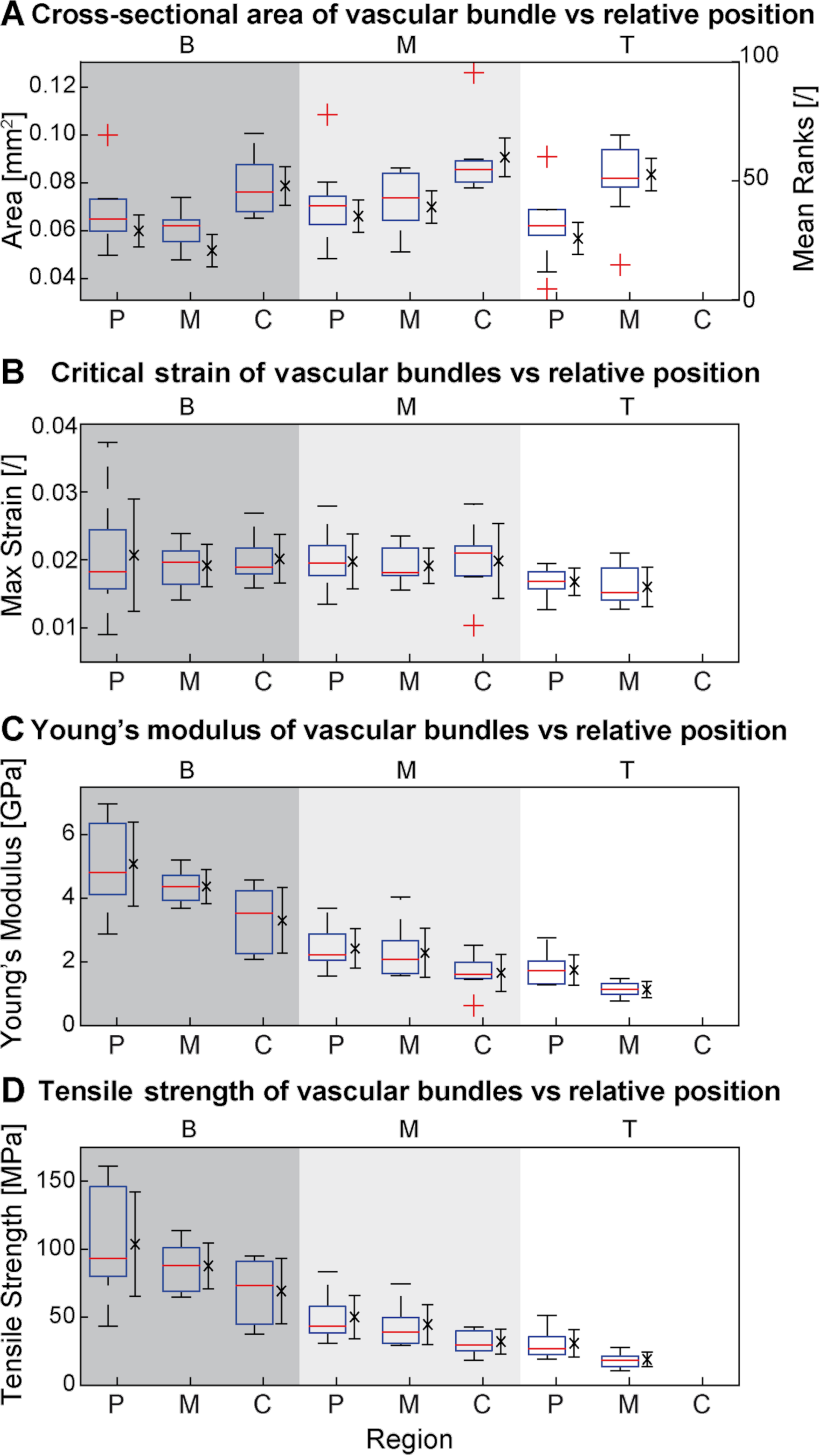
Morphometric data and material properties of vascular bundles of *Dracaena marginata*. (A) Cross-sectional area, (B) critical strain, (C) Young’s modulus and (D) tensile strength of vascular bundles vs relative radial position (periphery (P), middle (M) and centre (C)) and axial position (basal (B, dark grey area), middle (M, light grey area) and top (T, white area)) within the stem. In A to D, the full range of data is visualized by a boxplot of medians, quartiles (box), extreme values (whiskers) and potential outliers (+). In addition, in A the mean ranks (×) and standard error (whiskers) are plotted as error-bar plot; whereas in B to D the mean (×) and standard error (whiskers) are plotted as error-bar plot.

Figure 6A–D shows cross-sectional area, critical strain, Young’s moduli and tensile strength of individual vascular bundles of *D. marginata* for relative vertical (B,M,T) and relative radial positions (C,M,P) (see Supporting Information File 3 – Raw data and Descriptive statistics). The box–whisker plots show the range of measured values. In case of the cross-sectional area (Figure 6A) of the vascular bundles not all groups showed normal distribution, therefore the statistics were computed by Kruskal–Wallis test on mean ranks and only three groups differed significantly (chi-squared = 25.00, 7, *P* less than 0.001; see Supporting Information File 3 - Inferential statistics). No significant differences in group means were obtained for critical strain (Figure 6B) of the vascular bundles, where normal distribution was not rejected and the multiple comparisons was based on an ANOVA on means (ANOVA; F_7,66_ = 1.36, *P* = 2.3) and a post hoc test with Bonferroni adjustment. The normality assumption was again not rejected for the measurements of Young’s modulus (MoE) (ANOVA; F_7,66_ = 32.20, *P* less than 0.001) and tensile strength (ANOVA; F_7,66_ = 22.35, *P* less than 0.001) (Figure 6C,D respectively) and significant differences were observed between some groups for both strength and MoE. (We refer to Supporting Information File 3 – Inferential statistics, because the differences are too complex to show in Figure 6). Both strength and Young’s modulus show a similar pattern as previously described for bulk tissue axial Young’s moduli and density. For both mechanical properties a marked increase can be observed in radial direction from centre to periphery and a decrease in vertical direction from base to top. No measurements were available for the top-centre position, as the preparation of the samples failed to yield fibres suitable for measurement. The Young’s modulus (MoE) of all tested vascular bundles has a mean value of 2.77 ± 0.69 GPa.

### 4 Comparison of measured and calculated axial Young’s moduli in *Dracaena marginata* using the Voigt Model

**Figure 7:**
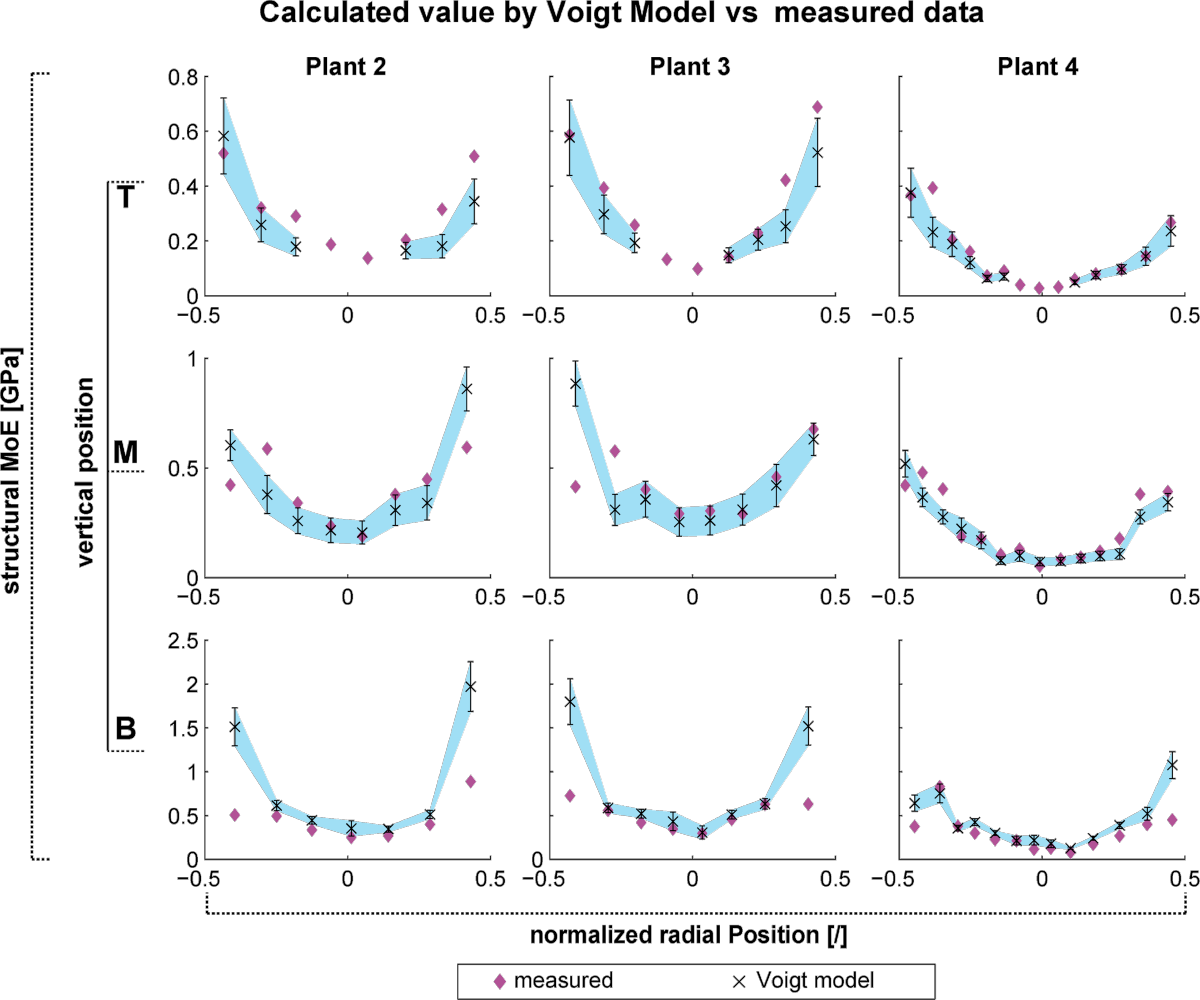
Measured vs calculated values (Voigt model) of the Young’s modulus of *Dracaena marginata*. Measured values are indicated by diamonds. Values calculated by using the Voigt model are depicted by mean values (×) and their standard deviation (indicated by the whiskers). The range of calculated values is furthermore visualized by the blue area, which represents a linear interpolation between mean values and their standard deviation.

Figure 7 shows the axial Young’s modulus calculated by the Voigt model compared to the measured values for bulk tissues (see also Figure 4) for plants 2–4 (see Supporting Information File 4 – Calculated data and Descriptive statistics). The calculated values for every sample are plotted as mean and standard deviation, whereas experimental measurements of the samples are plotted as diamonds. In the peripheral regions of the stem the values calculated via the Voigt model sometimes differ markedly from experimental measurements, but within central to middle regions the calculated values are in very good accordance with the bulk tissue measurements.

## Discussion

Due to the lack of sufficient literature data for the comparison of mechanical properties of *Dracaena* to other massive monocotyledon stems, we also compared our measured data with data from hollow culms of bamboo, horsetails and grasses as well as with massive herbaceous plants such as tobacco and with data from the wood of dicotyledons in order to present our data in a broad context.

### 1 Young’s modulus of five different monocotyledons

The significantly higher values of Young’s modulus of monocotyledons with rather small but potentially tree-like habit (*D. reflexa*), and a shrub-like habit (*Dracaena surculosa*) compared to the other three species with pronounced tree-like habit can be explained by two rationales. First, the stems of the tested specimen of *D. reflexa* and *D. surculosa* were very long and slender. These plants showed a much higher length-to-diameter (L/D) ratio (personal observation) than the investigated (other) tree-like monocots. At high L/D, the risk for Euler buckling increases and is met by the plants with an increased flexural stiffness via higher axial Young’s moduli.

A second important reason might be the advantage of a rather stiff stem for the ability to branch at wide angles, which is typically observed in *D. surculosa* and *D. reflexa*. Their wide-angled branchings appear often rather T-shaped (in *D. surculosa* more pronounced than in *D. reflexa*; compare Figure 1H with Figure 1G) while the narrow-angled branchings in *D. fragrans* and *D. marginata* appear rather Y-shaped (Figure 1D,E). It could be shown in [10] that the stress at rupture of the stem–branch connection and the “compactness” of the stem (i.e., many vascular bundles in the stem and a very distinct boundary between stem and branch) is higher in *Freycinetia insignis*, a plant with a branching morphology very similar to *D. surculosa,* than the stress at rupture and the “compactness” in *D. reflexa*. This might be mirrored in an axial Young’s modulus significantly higher in *D. surculosa* (similar to *Freycinetia insignis*) than in *D. reflexa*. It can also be hypothesized that the lack of such a “compactness” in the stem of *D. marginata* (see Figure 1A) and in *D. fragrans* [11–12] can be correlated to a lower Young’s modulus of the stems, and that this entails markedly smaller branching angles.

We also hypothesize that the high values of the axial Young’s modulus of *Dracaena surculosa* in rather young ontogenetic stages (median of axial Young’s modulus: 2.5217 GPa) are similar to the comparatively high stiffness in young palms that are mechanically “overbuilt” as documented in [8] as they cannot increase their flexural stiffness by secondary growth. This is also supported by the higher stress necessary for rupturing young branches of *F. insignis* [10], which lacks secondary growth as well. We assume that other monocots such as *Dracaena fragrans*, *D. marginata* or *D. reflexa* exhibit comparatively low Young’s moduli in young ontogenetic stages (median of axial Young’s modulus: 0.1799 GPa, 0.1235 GPa and 1.2090 GPa, respectively) due to their ability of secondary thickening. It allows for an increase in girth and formation of additional vascular bundles, which both contribute to a considerable increase in stiffness: The Young’s moduli in old stages of *Dracaena manii* of 4.9GP [13] surpass the other values of arborescent young *Dracaena* species with secondary growth by 5 to 10 times, while they are only approximately twice as high as that of (the assumably “overbuilt”) *D. surculosa*.

The comparatively low values of axial Young’s modulus measured for a rather old specimen of *Pandanus pygmaeus* can be explained by the fact that many Pandanaceae lack “compact” and thereby stiff stems and tend to produce aerial roots which support the stem and especially outgoing branches [14–15].

The two orders of magnitude difference between the axial and radial Young’s modulus indicate that the mechanical behaviour under axial tension is dominated by the vascular bundles [16] with a measured mean value of of 2.77 GPa in *D. marginata*. In contrast, the mechanical behaviour under radial tension is dominated by the parenchymatous tissue (Reuss model as described in [16] with a Young’s modulus of 0.003 GPa in *D. marginata*, (values for the Young’s modulus of parenchyma typically range between 0.001 and 0.003 GPa [17]). This assumption is further investigated via testing of individual vascular bundles and application of the Voigt model (see below).

### 2 Young’s modulus and density of stem samples in relation to radial and axial position in *Dracaena marginata*

The results of the spatial investigation of *D. marginata* indicate a strong dependence of the Young’s moduli on vertical and radial position (Figure 4) as well as on the density of the underlying tissue (Figure 5), which similarly has been demonstrated for palms [8,18] and more recently for Moso bamboo [2]. A multiple gradient in axial and radial direction has been shown here for density and Young’s modulus (Figure 4) with both parameters increasing towards stem base and periphery as in *Flagellaria indica*, a monocot climber, which stiffens towards the base and the periphery [19]. A similar gradient in radial direction has also been demonstrated for the tensile strength of tissues at various radial positions within the stem of Moso bamboo [20] and for the axial Young’s modulus of tissue strips in the culm of *Arundo donax* [21] as well as for the density in the oil palm *Elaeis guineensis* [22].

In *D. marginata*, an almost linear correlation of axial Young’s modulus and density was found. However, these observations are only valid for the measured ranges of density, an extrapolation beyond these ranges would yield great uncertainties due to the relative young age of the tested plants. Though many authors postulate a correlation between tissue density and Young’s modulus of a plant [23], in [24] it is argued that density alone is not sufficient for the evaluation of the Young’s moduli. For *D. marginata*, we hypothesize that an increase in tissue density may correlate (amongst others) with (1) the number of vascular bundles, (2) the area fraction of the vascular bundles, (3) the type of vascular bundles, (4) the cell wall composition [25], and (5) the number of cell-wall layers in vascular bundles or parenchyma and thereby also lignification and stiffness.

The values for density and Young’s modulus (Figure 5, mean value of 0.52 ± 0.39 GPa) are relatively low compared to other plants. The Young’s modulus in *D. marginata* increases linearly with density for both primary and secondary tissues. This general trend is also known for bamboo [2,26] and wood [27], though a linearity of the correlation – as found in *D. marginata* – was only found in older stems of the black locust (*Robinia pseudoacacia*) [28]. In palms similar values of longitudinal Young’s moduli are reported in fibre strips of thick-walled caps of vascular bundles tested under axial tension. These values ranged from 0.2 to 1 GPa in *Washingtonia robusta* [29], depending on the level of lignification. While we did not measure the degree of lignification in *D. marginata*, we assume that the density is a good indicator for the relative amount of lignification. As the density varies radially and longitudinally, a broad range of values in Youngs’ moduli from unlignified (0.03 GPa) to well-lignified (1.62 GPa) values was to be expected. This indicates that despite the very different growth strategies in palms (primary thickening) and *D. marginata* (secondary thickening), very similar mechanical properties can be achieved by lignification, which is also considered as a key factor in adjusting stiffness [29]. This postulated correlation of density, Young`s modulus and lignification is also substantiated by the similarity of value ranges for both density and Young’s modulus of *D. marginata* of 0.171 g·cm^−3^ and of *Iriartea* palms, another well-lignified palm [8] (Figure 5).

Similar values were measured also for the longitudinal Young’s modulus of tissue strips from internodes in *Equisetum giganteum* [30] and from internodes of *Equisetum hyemale* [31–32] as well as for the bending Young’s modulus measured in axes of herbaceous dicotyledon plants such as tobacco [33].

These values of approx. 1 GPa for horsetails and 0.8 GPa for tobacco are well within the range of values found in *D. marginata*. The similarity cannot be explained by a similar degree of lignification as extant horsetails as well as herbaceous dicotyledon plants are not or very poorly lignified and rely on the turgor of their parenchymatous tissues (additionally to strengthening tissues such as collenchyma or sclerenchyma) for providing stiffness. While a high turgescence of parenchyma surely also adds to the stiffness in *D. marginata* (as the parenchyma acts as a spacer tissue that keeps the stiff vascular bundles in place), the amount of relative contribution of turgor to stiffness cannot be quantified by the methods used in this study and necessitates further analyses.

The axial Young’s modulus of *D. marginata* with a mean value of 0.52 ± 0.39 GPa is markedly lower than that of the Moso bamboo with an MoE of 10.56 GPa [2]. It is also lower than the longitudinal Young’s modulus in culms of *Arundo donax* with values of 9–10 GPa measured with bending tests [34], and within the lowest range of 1 to 11 GPa found in tensile tests of tissue strips of *Arundo donax* [21].

This is to be expected as the density (about 0.630 g·cm^−3^ for an unspecified bamboo [9] (see Figure 5)) and the degree of lignification of bamboo is much higher than that of *Dracaena* species. This also applies to woody plants such as conifers and broad-leafed trees such as the eastern white pine with a density of 0.350 g·cm^−3^ and a Young’s modulus of 8.50 GPa as well as the Douglas fir, the white spruce and the northern oak with densities ranging from 0.360 to 0.630 g·cm^−3^ and Young’s moduli ranging from 9.6 to 13.4 GPa [35]. However, the age of these trees was much higher than that of *D. marginata*. It can be assumed that *Dracaenaceae* can also attain higher values in older plants.

### 3 Tests of the vascular bundles of *Dracaena marginata*

The presence of a high number of rather small (Figure 6A) vascular bundles in the periphery and a comparatively lower number of larger vascular bundles in the centre can be well discerned in cross-sections of *Dracaena marginata* (Figure 1A). It can be also observed in many other monocotyledon plants such as palms [29], Moso bamboo [2] and the giant reed *Arundo donax* [34]. It was suggested in other studies that this increases the second moment of area of mechanically relevant tissues and thereby increases the flexural stiffness of the stems [21,29,34,36–37]. We also propose a similar increase of flexural stiffness of the stems of *D. marginata*. Though we did not measure the flexural stiffness of these plants in our study, the twofold increase of number of vascular bundles and their tensile Young’s moduli towards the periphery both increase the second moment of area and the stiffness of mechanically important tissues so that the flexural stiffness of axes of *D. marginata* is also increased.

The lack of significant differences in the critical strain found for vascular bundles across the periphery and towards top or base (Figure 6B) can be explained by the similar structure of all vascular bundles so that no large differences in critical strain might be expected. This does not hold true, however, for the stress and the stress–strain ratios. These are very different in peripheral and radial positions and lead to very different Young’s moduli (Figure 6C) and tensile strength of the vascular bundles (Figure 6D).

The Young’s modulus of the vascular bundles with values ranging from mean values of 5.08 ± 1.33 in peripheral bundles at the stem base down to 1.74 GPa ± 0.48 GPa is largely overlapping the range of approx. 4.3 to 0.5 GPa reported for strips of the hypodermal sterome in *Equisetum hymale* [32]. This indicates that despite the fundamentally different developmental growth and systematic position of *D. marginata* and *E. hyemale*, the two plants respond to similar mechanical constraints imposed by the self-supporting growth habit by developing strengthening tissues with similar mechanical properties at the outermost periphery of their axes.

The Young’s moduli of the vascular bundles in *D. marginata* are noticeably higher than that of fibre caps in the palm *Washingtonia robusta* measured for which values were approx. 0.4 to 0.5 GPa for fibre strips close to the phloem [38]. We assume that this is due to the very low level of lignification of these fibre strips in *W. robusta*. The values found for fresh bundles in palms and dragon trees are markedly lower than the value of 36 GPa measured for the Young’s modulus in air-dry fibres of Moso bamboo [39], which represents one of the few values reported for other monocot bundles. We propose the same rationale as given for the tissues and attribute the higher values found in Moso bamboo to a higher level of lignification in bamboo as compared to *D. marginata*.

The increase of tensile Young’s modulus (stiffness) of the vascular bundles is well correlated with the increase of the Young‘s modulus of the tissues. The twofold gradients, i.e., stiffer towards the outside and toward the base is also observed for the Young’s moduli of the respective vascular bundles. This holds also true for the tensile strength of the vascular bundles. A single (radial) gradient for the tensile Young’s modulus and tensile strength of vascular bundles was also found across the culm wall in Moso bamboo [39].

Finally, the comparably high tensile strength of the vascular bundles at the periphery of the trunks is of high importance for stabilizing the stem–branch attachments as these are mainly supported by (the fibrous parts of) vascular bundles under tensile stress [40]. The absolute values with a mean of 55 ± 17 MPa are well below the values reported for bamboo which range from 810 MPa [4] for fresh samples to 550 MPa [39] for dry samples as well as 610 MPa [3,41] for samples of which the moisture content remains unclear. It can be stipulated that values for vascular bundles in the region of stem–branch attachments in *D. marginata* (which we did not measure) can also assume higher values as a mechanical response to higher load stresses imposed by lateral branches.

### 4 Comparison of measured and calculated longitudinal Young’s moduli of tissues in *Dracaena marginata* using the Voigt model

The calculated values for the Young’s modulus of the tissue using the Voigt model match the data for the measured values in *Dracaena marginata* very well except for the outermost peripheral positions in the stem (Figure 7). There, the data are sometimes markedly overestimated (basal position of plants) or slightly underestimated (top positions of plants) or both, i.e., slightly over- or underestimated (middle positions of the plants). The Voigt model depends upon three factors: (1) the Young’s modulus of the ground tissue, (2) the Young’s modulus of the vascular bundles, and (3) the volume fractions of ground tissue and vascular bundles. As we attributed a constant value to the Young’s modulus of the ground tissue, the calculated values of our Voigt model can only change due to variation of the last two factors. The marked overestimation of the Young’s modulus cannot be explained by the peripheral increase of the Young’s modulus of the vascular bundles, which increases from centre to periphery approximately by a factor 1.5 (see Figure 6C), an augmentation which is in good accordance with the increase of the measured values for the Young’s modulus of the tissues. Possible causes for the overestimation are drying and stiffening of the vascular bundles during testing or/and an overestimation of the volume fraction of the vascular bundles. Another possibility is the assumption (for the Voigt model) that the vascular bundles are perfectly arranged in parallel to the axis of the stem, which they are not. In fact, it was shown by Tomlinson and others that the intertwining course of the vascular bundles in *Dracaena* [11–12] and other monocotyledon stems [42–46] is highly complex and characterized by many anastomoses. The deviation of the vascular bundles from an idealised axial arrangement would also lead to an overestimation of the values calculated by the Voigt model.

## Conclusion

The comparably high values of axial Young’s modulus in tissues of the young shrub-like plants *Draceana surculosa* and *D. reflexa* are interpreted as a response to the increased “need” for higher bending stiffness of (1) their slender stems and (2) in the regions of stem branch–attachment. This is because *Draceana surculosa* and *D. reflexa* branch at higher branching angles than young tree-like *D. marginata* and *D. fragrans*, which have a thicker stem and branch with rather narrow angles. Moreover, the lack of secondary growth is hypothesized to lead to a mechanical “overbuilding” of young axes as present in palms [8]. It would have been worthwhile to analyse whether a consequent “underbuilding” of old axes as in palms [8] is present in *D. surculosa* and *F. insignis*. This is however prohibited by the absence of sufficiently old ontogenetic phases in *D. surculosa* and F*. insignis* as the entire shoot is replaced after inflorescence.

Results prove an axial and radial gradient of mechanically relevant properties in the stems of *D. marginata*. It could be shown that towards the base and the periphery of the stems there is an increase of density (as described for palms [8]) and of the axial Young’s modulus of tissues as described in the radial direction for Moso bamboo [2].

While the values for density can reach the values of other monocotyledons such as bamboo, the axial Young’s modulus is generally one order of magnitude lower [2]. This is interpreted as a consequence of the higher lignification of bamboo stems. The abundance of wood is also interpreted as one of the causes of the higher values for the axial Young’s modulus in conifers and dicotyledonous trees such as Douglas fir, white spruce and northern oak [35] as compared to stems of *D. marginata*. Another reason is the less dense arrangement of the fibrous bundles in an atactostele in *D. marginata* and the dense arrangement of stiff tissues in conifers and dicotyledons. Relationships between density and the axial Young’s modulus are visualized in a material property chart (Figure 5) and thereby fill major “white spots” for biological material properties.

The two-dimensional gradient as present for the axial Young’s modulus of the tissues in *D. marginata* is also mirrored by a gradual increase of the Young’s modulus and the tensile strength of the vascular bundles towards the base and the periphery. The Young’s modulus of the vascular bundles is approx. five times higher than the Young’s modulus of the bulk tissue, so that the measured values for the longitudinal Young’s modulus of the tissues (first hierarchical level) can be assumed to be dominated by the values of the longitudinal Young’s modulus of the vascular bundles (second hierarchical level). This assumption is verified by the good accordance of the calculated values of the axial Young’s modulus via the Voigt model.

The higher strength of fibrous bundles in the periphery is also vital for the support of (peripherally developing) branches in *D. marginata*.

## Biomimetic approaches and outlook

The values for the Young’ s modulus for the five tree- or shrub-like monocotyledons, the density–stiffness gradients along the axis of *D. marginata* and the values for the tensile strength and Young’s modulus of the vascular bundles add to the knowledge and help to understand the functional anatomy and the biomechanics of arborescent monocotyledons. They also confirm the status of these plants as interesting concept generators for the development of branched and unbranched fibre-reinforced materials and structures with enhanced properties [6,47–49]. The axes of these plants consist of materials that combine low density with sufficient mechanical stiffness, which result in lightweight structures with optimized structural density–stiffness gradients. Additionally, the differentiation of the stem in ground tissue and vascular bundles is very similar to technical fibre-reinforced materials consisting of stiff fibres embedded in a more flexible matrix. Finally, the technical implementation of the functional principles of such plants can be aided by finite element modelling [7]. Further studies using in vivo magnetic resonance imaging allow for revealing the internal stress–strain relationships in mechanically loaded stems of monocotyledons [50] and shall be extended to other plants.

## Experimental

### Materials

Mechanical testing was performed on five different tree- or shrub-like monocotyledon species. Of the Dracaenaceae, three tree-like species with the potential for secondary growth, *Dracaena marginata*, *Dracaena fragrans* and *Dracaena reflexa*, as well as a shrub-like species, *Dracaena surculosa*, were tested. In addition, another tree-like monocotyledon without secondary growth, *Pandanus pygmaeus* (Pandanaceae), was also tested. All tree-like Dracaenaceae were purchased from commercial nurseries and cultivated in the Botanic Garden of the University of Freiburg. *D. surculosa* was also cultivated in the Botanic Garden Freiburg, whereas *P. pygmaeus* was cultivated at the Botanic Garden of the Technical University Dresden.

### Methods

For measuring of the material properties three sets of experiments were performed: (1) Tensile tests on the stem tissue of all five species (Figure 2A), (2) a detailed tensile analysis of *D. marginata* tissues with respect to relative radial (Equation 1) and axial position (top, middle, base) within the plant stem (Figure 2B), and (3) a detailed tensile analysis of the vascular bundles of *D. marginata* also with respect to relative radial and axial position within the plant stem (Figure 2C). In (1) only the Young’s moduli of the plants were assessed. In (2) material density, Young’s moduli, water-content and area-fraction of vascular bundles of the samples were determined in a similar way as the methods described in [2]. In (3) the Young’s moduli, the tensile strength and the critical strains were calculated.

### Young’s modulus of five different monocotyledons (1)

#### Testing procedure and determination of Young’s moduli and strains

The axial (along the stem axis) as well as the radial Young’s moduli of the five tree- or shrub-like monocots were measured under tensile stress by using two universal testing devices. An Instron^®^ 4466 device (Instron, Norwood, Massachusetts, USA; retrofitted by Hegewald & Peschke, Nossen, Germany) was used for (1) *D. fragrans* (16/17 samples (axial/radial) from 5 plants), (2) *D. marginata* (46/37 samples from 10/9 plants), (3) *D. reflexa* (8/— samples from one plant) and (4) *D. surculosa* (13/— samples from one plant), and a Zwick-Roell Z250 (Zwick-Roell AG, Ulm, Germany) was used for (5) *P. pygmaeus* (10/8 samples from one plant (see Supporting Information File 1 – Raw data). Due to the low radial dimension of *D. reflexa* and *D. surculosa*, a test in radial direction was not feasible. Samples of varying size were cut from the stems and fixed in the clamps of the testing device (Figure 2A). The compressive force of the clamping jaws was carefully adjusted to prevent radial crushing of the sample but at the same time to prevent any axial slip during testing. Details on the individual testing procedures per plant are provided in Supporting Information File 5.

For the determination of the Young’s modulus (MoE) an ordinary least squares fit (OLS) was performed with the Matlab (2014a, The Mathworks Inc., Natick, Massachusetts, USA) routine ‘LinearModelFit’ on the linear section (selected manually) of the stress–strain curve of each individual measurement (Figure 2A). The slope of the OLS corresponds to the Young’s modulus of the sample. The tensile stresses (σ_Z_) were calculated by dividing the measured force (*F*) by the cross-sectional area (*A*_cross_) of the sample:

[2]
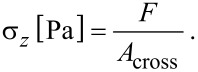


The strains were calculated by dividing the measured displacements (Δ*l*) by the free length of the sample (*l*_free_):

[3]
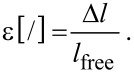


#### Descriptive and inferential statistics

For each plant species the descriptive statistics for the MoE, including mean, standard error of the mean (SE), standard deviation (STD), median, quartiles, interquartile range (IQR) and a Lilliefors test for normal distribution (H0: normal distribution), were computed in axial and radial direction (see Supporting Information File 1 – Descriptive statistics). An inter-species comparison was calculated in axial direction by one-way-ANOVA (H0: equality of means at α = 0.05) and a post hoc multiple comparison on means (Bonferroni adjustment, see Supporting Information File 1 – Inferential statistics). The results for the axial Young’s modulus were plotted as a combination of a box–whisker plot indicating medians, quartiles, interquartile range and extreme values and an error-bar plot of means and standard error for each of the five tested plants (Figure 3A). As the Young’s moduli in radial direction were not normally distributed (Lilliefors test) for all plants, an inter-species comparison was performed by a Kruskal–Wallis ANOVA on mean ranks (H0: equality of mean ranks at α = 0.05) with a post hoc multiple comparison on mean ranks (Tukey–Kramer, see Supporting Information File 1 – Inferential statistics). The results for the radial Young’s moduli were plotted as a combination of a box–whisker plot and an error-bar plot of the mean ranks for each plant species except for *Dracaena reflexa* and *D. surculosa* for which no mechanical measurements could be performed (Figure 3B). Significances on the 5% level are displayed in letter grouping, whereby identical letters group data with no significant difference.

### Young’s modulus and density of stem samples in relation to relative radial and relative vertical position in *Dracaena marginata* (2)

#### Sample preparation and testing procedure

For a more detailed investigation of the Young’s moduli within the selected representative species, five specimens of *D. marginata* were used. A total amount of 152 samples were collected in five different axes. 109 samples originate from four stems below branchings (termed plants 1–4), and 43 samples from a first-order branching (termed plant 5). The stems were divided into three axial zones in order to compare the Young’s modulus in different tissue regions of the specimens. Out of each axial zone one cylindrical sample was taken (Figure 2B). Samples from the lower third of the sample were termed B (for basal zone), those in the intermediate part were termed M for (middle zone) and those in the upper third were termed T (for top zone). This axial subdivision could not be performed in the first order branch.

Subsequently, out of each cylindrical sample for B, M and T a long median rectangular section with the following dimensions was cut (Figure 2B): a (transverse) thickness *x* equal to the radial width of the stem in one direction, a depth *y* between 5 and 7 mm in the perpendicular direction, and a length *z* (between 7 and 10 mm) for the basal, middle or top part of the stem. As a result, three such median sections (B, M and T) originated from each of the four stems. In the first-order branch, one median rectangular section (termed 5A) was cut from the cylindrical sample of the branch as described above (Figure 2B) but an additional second tangential rectangular section (adjacent to the median section and termed 5B) was produced in order to gain more data on secondary vascular tissue. The data of the median section (5A) were used for the assessment of the regional dependency of the tissue density and Young’s modulus (MoE) in Figure 4. The combined data of both sections (5A and 5B) were used for regression analysis of the density dependence of the MoE and in the materials property chart (see below for details).

To determine the radial variation of the material properties for each zone and plant, the original rectangular samples were then cut into smaller rectangular samples along the length and perpendicular to the width of the original sample (Figure 2B). Each sample now represents a radial position (P, M or C) within a defined axial zone (B, M, T) and plant. Despite the great care laid on producing regular rectangular samples with a defined radial width, a variation of the radial width (*w*) of the different cut samples between 1.13 and 2.90 mm (mean of 1.86 ± 0.39 mm) was inevitable because of deflections of the cutting knife due to the inhomogeneity of the stem tissue.

Each sample was then measured in tension with a universal testing device Instron^®^ 4466 (Instron^®^, Norwood, Massachusetts, USA; retrofitted by Hegewald & Peschke, Nossen, Germany). To prevent slipping of the samples, they were fixed with all-purpose glue (Blitzschnelle Pipette, UHU GmbH & Co KG, Bühl, Germany) in four L-shaped aluminium brackets which then were clamped to the jaws of the testing device (Figure 2B). The free length (*l*_free_) for each sample, which ranges from 14.6 to 26.3 mm, is important for the later calculation of strains and is documented in Supporting Information File 2 – Raw data. More details about the testing procedure (e.g., load capacity of the force transducer and boundary conditions of the test) are provided in Supporting Information File 5.

In order to obtain dry density, water content and cross-sectional area as well as the number and area of vascular bundles for each of the mechanically tested samples, the samples were subsequently cut in one large and one small subsample. The small sample was embedded in PEG 2000 and thin sections of 25 µm thickness were produced with a microtome. Microscope images (Olympus BX61 microscope, Olympus Corp., Tokyo, Japan) of the cross-sections were then evaluated for cross-sectional area (*A*_cross_) of the sample, number of vascular bundles (#_vb_) and total area of vascular bundles within the sample. Based on these images, it was also possible to determine visually if primary or secondary vascular tissue was predominant. The larger subsample was measured in length (*l*_sub_, to calculate the fresh volume (*V*_fresh_)) and weighted on an electronic microscale (Mettler UMT2, Mettler-Toledo Ltd., Leicester, United Kingdom) to obtain the fresh mass (*M*_fresh_). Subsequently, the sample was oven-dried at 60 °C for four days and the oven-dried mass (*M*_dry_) was measured.

#### Data analysis and statistics

Young’s Moduli and strains were computed from the slope of the second load cycle (Figure 2B) by the same methods described above for experiment (1) in Equation 2 and Equation 3.

The volume (*V*_fresh_) of each fresh subsample was calculated by multiplying the subsamples length *l*_sub_ with the measured cross-sectional area (*A*_cross_):

[4]
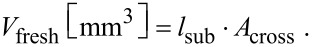


The dry density (ρ_dry_) was then calculated by dividing the oven-dried mass (*M*_dry_) by the fresh volume (*V*_fresh_) of the sample:

[5]
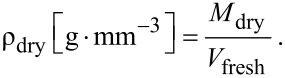


The water content (*WC*) is the difference between the fresh mass (*M*_fresh_) and the oven-dried mass (*M*_dry_) divided by the fresh mass (*M*_fresh_):

[6]
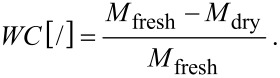


The relative radial position of each sample was calculated (for all axial zones of all five plants) in order to be able to compare radial differences in axial Young’s moduli and to assess if the Young’s moduli in the centre of the stem (C, see Figure 4) differ from that in the periphery (P) or in the intermediate zone (M). The radial position of each sample, i.e., the radial distance from the centre of the stem, was calculated by using Equation 7 and Equation 1, where *w_i_* is the radial width of each sample.

Diameter of stem section (*D*_sect_):

[7]
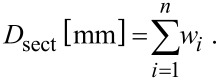


Radial position of samples (*P*_rad_):

[1]
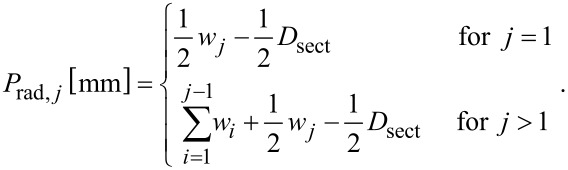


For the determination of the relative radial position (C, M and P), the data of the radial position is normalized by the diameter of the stem (Equation 8) and grouped according to Equations 9–11.

Normalized radial position of samples (*P*_norm_):

[8]
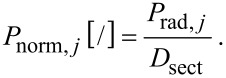


Relative radial position [grouping]:

[9]
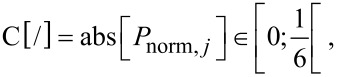


[10]
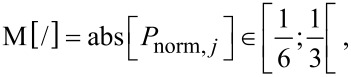


[11]
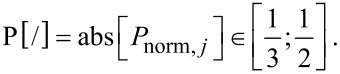


In Figure 4 the dry density (ρ_dry_) and the Young’s modulus (MoE) are plotted versus the normalized radial position of each sample for all five plants. The ranges of the relative radial positions (C, M, P) are indicated by the intensity of the grayscales of the background in each graph.

For the evaluation of the relationship of MoE and ρ_dry_ an ordinary least squares fit (OLS) was performed with the Matlab (2014a, The Mathworks Inc., Natick, Massachusetts, USA) routine ‘LinearModelFit’. The data (MoE and ρ_dry_) of all five plants (a total of 152 samples) was linearized by log_10_–log_10_ transformation (analogous to [8]) and grouped as primary and secondary tissues. As a linear regression model, the MoE was assessed with an interaction term of ρ_dry_ and tissue category (log_10_(MoE) ≈ 1 + log_10_ρ_dry_·tissue) returning a regression table with the statistics of the model (see Supporting Information File 2 - Linear regressions). An ANOVA was calculated to determine whether the slopes of primary and secondary tissues differed (see Supporting Information File 2 - ANOVA). The data for dry density and Young’s modulus were integrated in a materials property chart (Figure 5).

### Tests of the vascular bundles of *Dracaena marginata* (3)

#### Sample preparation and test procedure

The axial and radial positions of the rectangular samples were defined as for the stem tissues (see previous set of experiments). Across the diameter of the stem we obtained five rectangular samples (Figure 2C). These rectangular samples were then squeezed laterally to destroy parenchymatous cells and then submerged in H_2_O_dest_ for the days in order to further macerate the parenchymatous tissue. We obtained a loose network of vascular bundles (Figure 2C). Individual bundles could then be separated by manual extraction (Figure 2C). By carefully pulling the bundles through two fingers, most residual parenchymatous tissue could be removed from the surface of the bundles. Each bundle was then fixed on two aluminium plates (Figure 2C) at a distance of roughly 10 mm with all-purpose glue (Blitzschnelle Pipette, UHU, GmbH & Co KG, Bühl, Germany). Samples were kept in a box with wet paper tissue until testing to prevent the samples from drying.

The tensile testing of the samples was performed at a constant speed of 5 µm/s until failure of the samples on a custom-made tension–compression testing device equipped with a load cell of 50 N maximal force. The free length of the samples was measured as the distance between the plates. Displacements until the first increase in force (distance Δ*x* in Figure 2C) were subsequently added to the free length of the samples. The cross-sectional area of the vascular bundles was calculated using the diameter of the vascular bundles measured in images taken by a binocular (Olympus SZX 9, Olympus Corp., Tokyo, Japan) equipped with a digital camera. The images were taken from remaining ends of the vascular bundles after mechanical testing. The cross-sectional area was assumed as circular and constant over the length of the sample, an assumption that holds true in good approximation as proven by visual inspection of the undamaged vascular bundles. These test results allow for the determination of Young’s modulus, critical strain (ε_crit_) and tensile strength (σ_max_) of the vascular bundles (see Supporting Information File 3 - Raw data).

#### Data analysis and statistics

For the determination of the Young’s moduli an ordinary least squares fit (OLS) was performed as described above for the experiments in set (1). Critical strain is the strain at rupture at a critical force, and tensile strength is the stress at the critical force applied to the sample. The descriptive statistics for area, Young’s modulus (MoE), critical strain and tensile strength were calculated as described above for experimental set (1) and are provided in Supporting Information File 3 – Descriptive statistics. As the results for the area were not normally distributed (Lilliefors test) for all groups, an inter-species comparison was performed by a Kruskal–Wallis ANOVA on mean ranks (H0: equality of mean ranks at α = 0.05) with a post hoc multiple comparison on mean ranks (Tukey–Kramer, see Supporting Information File 3 – Inferential statistics). The results for the area were then plotted as a combination of a box–whisker plot indicating medians, quartiles, interquartile range and extreme values and an error-bar plot of mean ranks and standard error for each corresponding group/tissue zone (Figure 6A). Due to the normality of critical strain, MoE and tensile strength, a comparison between groups was calculated by one-way ANOVA (H0: equality of means at α = 0.05) and a post hoc multiple comparison on means (Bonferroni adjustment, see Supporting Information File 3 – Inferential statistics). The results for critical strain, MoE and tensile strength were plotted as a combination of a box–whisker plot indicating medians, quartiles, interquartile range and extreme values and an error-bar plot of means and standard error for each of the corresponding group/tissue zone (Figure 6B–D).

### Comparison of measured and calculated longitudinal Young’s moduli of tissues in *Dracaena marginata* using the Voigt model (4)

In composite theory, an upper bound modulus for loading parallel to the fibres can be estimated by the rule of mixture, more precisely the Voigt Model [51]. It has been shown that this can also be applied to plant tissues [16] and to entire plant stems [49,52–53]. Hence, the plant bulk tissues (fibres and parenchyma) of *Dracaena marginata* are approximated to axially parallel, non-branching fibres embedded in a parenchymatous matrix. Here, the calculated Young’s modulus of the bulk tissues MoE_t_ is the result of the Young’s modulus of the vascular bundles (MoE_vb_) and the Young’s modulus of the ground tissue (MoE_gt_) set in proportion to their volume fraction (Equation 12):

[12]
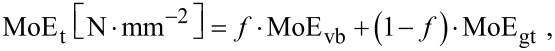


where *f* is the volume fraction of the vascular bundles and is given by:

[13]
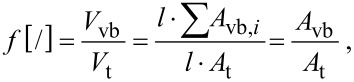


with *V*_vb_ being the cumulative volume of all vascular bundles and *V*_t_ the volume of the bulk tissue. As the length *l* is a constant factor for both fibre and tissue, the volume fraction can be reduced to the area fraction of the cross-sections with *A*_vb_ being the cumulative cross-sectional area of all vascular bundles (*A*_vb_*_,i_*) and *A*_t_ the cross-sectional area of the bulk tissue.

For every measured sample of plants 2–4, the bulk tissue MoE_t_ was calculated, therefore the data evaluated by the methods described for the experiment sets 1–3 are put together in the following manner: The mean value of the radial Young’s modulus of *D. marginata* (0.0033 GPa, set 1) has been assigned to the ground tissue (MoE_gt_) because it can be assumed that these values are dominated by the ground tissue (Reuss model, see further elaborations in the Discussion section). Assignment/mapping of tissue data (see set 2) and fibre data (see set 3) has been made possible by the grouping of the tissue and fibre data in axial (B, M, T) and radial (P, M, C) zones. For every tissue sample measured (set 2), the cross-sectional area of the sample (*A*_cross_) has been assigned to the cross-sectional area of the bulk tissue *A*_t_. The cumulative cross-sectional area of the vascular bundles (*A*_vb_) can be further described by the product of number of vascular bundles within a tissue cross-section (#_vb_, result from methods in set 2) and the cross-sectional area of the vascular bundles (*A*_vb,_*_i_*):

[14]
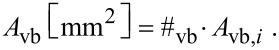


Here, the simplification of a homogenous size of vascular bundles within each cross-sectional area has been made.

The number of calculations using the Voigt model (Figure 7) for each of the nine tissue zones –crosswise combinations of the tree axial zones, basal (B), middle (M) and top (T), with the three radial zones, periphery (P), middle (M) and centre (C) – correspond to the number of tissue samples in the tissue zones (Figure 4) multiplied by the number of measured vascular bundles in the assigned zone. In total 782 calculations have been made (see Supporting Information File 4 – Calculated data w. Voigt model).

For each zone, the calculations have been assessed by descriptive statistics (mean, STD, standard error of the mean, see Supporting Information File 4 – Descriptive statistics). The mean and standard deviation has been plotted versus the relative position for each plant individually in Figure 7. In addition, the measured value of each sample was added to the plot for comparison. If the values of the measured data are within the range of the standard deviation of the calculated data using the Voigt model, we consider the Voigt model as valid, i.e., able to adequately match the calculated with the measured Young’s modulus of the tissue.

We use the Voigt model in the present study to test whether the measured values for the longitudinal Young’s model of the tissues (first hierarchical level) are dominated by the values of the longitudinal Young’s modulus of the vascular bundles (second hierarchical level).

## Supporting Information

File 1 Raw data of measurements and statistics for stem segments of various monocotyledons.

File 2 Details on testing procedures of stem segments for various monocotyledons.

File 3 Raw data of measurements and statistics for stem segments of *Dracaena marginata.*

File 4 Raw data of measurements as well as statistics for individual vascular bundles of *Dracaena marginata*.

File 5 Data and statistics using Voigt’s model for assessing the structural Young’s modulus of *Dracaena marginata*.
